# Association of serum periostin with bone microarchitecture, muscle mass and function in Chinese postmenopausal women

**DOI:** 10.3389/fendo.2026.1808866

**Published:** 2026-04-07

**Authors:** Yiyi Gong, Yushuo Wu, Xiaosen Ma, Lijia Cui, Xiang Li, Qianqian Pang, Yue Chi, Ruizhi Jiajue, Wei Liu, Ou Wang, Mei Li, Xiaoping Xing, Zaizhu Zhang, Wei Yu, Yan Jiang, Weibo Xia

**Affiliations:** 1 Department of Endocrinology, Laboratory of Endocrinology, National Commission of Health, State Key Laboratory of Complex Severe and Rare Diseases, Peking Union Medical College Hospital, Chinese Academy of Medical Sciences and Peking Union Medical College, Beijing, China; 2 Center for Biomarker Discovery and Validation, National Infrastructures for Translational Medicine (PUMCH), Institute of Clinical Medicine, Peking Union Medical College Hospital, Chinese Academy of Medical Sciences and Peking Union Medical College, Beijing, China; 3 Department of Endocrinology, Beijing Tsinghua Changgung Hospital, School of Clinical Medicine, Tsinghua University, Beijing, China; 4 Department of Radiology, Peking Union Medical College Hospital, Chinese Academy of Medical Sciences and Peking Union Medical College, Beijing, China

**Keywords:** bone microarchitecture, fall, fracture, muscle, periostin, postmenopausal women

## Abstract

**Purpose:**

High serum periostin is linked to an increased risk of osteoporotic fractures in postmenopausal women. However, the relationships between serum periostin and bone microarchitecture, particularly muscle mass, and muscle function remain unclear. This study aims to investigate the relationships between serum periostin and bone mineral density, bone microarchitecture, muscle mass and function, falls, and fractures in community-dwelling Chinese postmenopausal women.

**Method:**

Serum periostin levels were measured in 135 Chinese postmenopausal women. Dual-energy X-ray absorptiometry assessed areal bone mineral density (aBMD), lumbar trabecular bone score (TBS), and muscle mass. High-resolution peripheral quantitative computed tomography (HR-pQCT) evaluated bone microarchitecture. Muscle function and fall risk were assessed using handgrip strength, the Short Physical Performance Battery (SPPB), the Timed Up and Go (TUG) test, and the Falls Risk for Older People in the Community (FROP-Com) screening tool.

**Result:**

The mean serum periostin was 1770.3 ± 440.4 pmol/L. Serum periostin was negatively correlated with bone microarchitecture parameters, total area (Tot.Ar), cortical perimeter (Ct.Pm), trabecular area (Tb.Ar), estimated bone strength stiffness, and failure load. Negative correlations were also found with appendicular skeletal muscle mass (ASM), balance test scores, and gait speed. Conversely, serum periostin was positively correlated with TUG time and FROP-Com scores.

**Conclusion:**

Higher serum periostin is associated with smaller bone cross-sectional area, lower estimated bone strength, reduced muscle mass, and higher fall risk, indicating its potential as a predictor for assessing skeletal and muscular health in postmenopausal women.

## Introduction

Osteoporosis is a systemic skeletal disease characterized by reduced bone mass, deterioration of bone microarchitecture, impaired bone quality, and an increased risk of fracture (1, 2). According to nationwide epidemiological studies in China, the prevalence of osteoporosis among individuals aged 50 and above was 19.2%, with a higher prevalence of 46.4% for osteopenia (3). Sarcopenia, defined as the progressive and generalized loss of skeletal muscle mass and function, is another common age-related condition (4–6). Both osteoporosis and sarcopenia are strongly associated with aging and contribute to a higher risk of falls, fractures, hospitalization, and mortality (7–11). Therefore, it is crucial to not only focus on the bone health but also pay attention to the muscle health (7–11).

Periostin is a soluble extracellular matrix protein mainly expressed in the periosteum, where it plays an important role in regulating osteoblast proliferation and differentiation (12). In mouse models, deletion of the periostin gene led to reduced bone density, thinner cortical bone, and decreased bone strength (13). Several studies have examined the association between serum periostin and areal bone mineral density (aBMD) (14–18), and some have reported links between circulating periostin levels and the risk of osteoporotic fractures (19, 20). Beyond bone, periostin is also involved in the regulation of connective tissues (21, 22). Evidence from Postn^-^/^-^ mouse models indicates that periostin is involved in skeletal muscle remodeling, and its deficiency is associated with reduced muscle fibrosis as well as improved muscle structure and function (23, 24).

Therefore, when exploring the regulatory role of periostin in osteoporosis, it is essential to consider its multifaceted role. While extensive literature exists on the association between periostin and bone metabolism, studies concurrently assessing periostin’s relationship with bone microarchitecture and muscle function in postmenopausal women are still scarce. This study aims to investigate the relationship between serum periostin and bone microarchitecture, muscle mass, muscle function, risk of fall and fractures in community**-**dwelling Chinese postmenopausal women.

## Subjects and methods

### Subjects and study design

This was a cross**-**sectional study in community**-**dwelling postmenopausal women based on the Beijing subgroup of the Chinese Vertebral Osteoporosis Study (ChiVos). 138 participants were enrolled from September 2021 to December 2021. The inclusion criteria were as follows: (1) age more than 50 years old; (2) the history of living in a Beijing urban community for over a half year; (3) no menstruation for at least one year by self**-**reporting or at least six months after bilateral oophorectomy. Exclusion criteria included: (1) non Asian; (2) cognitive impairment or physical dysfunction. The study adhered to the principles of the Declaration of Helsinki and was approved by the Ethics Committee of Peking Union Medical College Hospital (JS**-**2905). All participants were provided with full information about the study and signed informed consent forms. Only 135 participants whose serum periostin levels were measured were enrolled in this study. Based on the distribution of serum periostin levels in the cohort, participants were categorized into three groups in ascending order: T1 (850.9–1530.5 pmol/L), T2 (1549.9–1956.6 pmol/L), and T3 (1968.7–2733.2 pmol/L).

### Clinical information collection

General information was collected by the interviewer administered questionnaire (25). This information included the history of falls and fractures. Falls were reported by participants and fractures were confirmed using X**-**rays. Measured and recorded height and weight of the participants and then calculated body mass index (BMI).

### Biochemical measurements

Serum calcium, phosphate, and alkaline phosphatase (ALP), anine aminotransferase (ALT), creatinine (Cr) were measured by an auto**-**analyzer (Beckman Coulter AU5800, USA). Parathyroid hormone (PTH) was detected by an autoanalyzer (Beckman Coulter DXI800, USA). 25**-**hydroxyvitamin D [25(OH)D], procollagen type 1 N**-**terminal propeptide (P1NP), C**-**terminal cross**-**linking telopeptide of type I collagen (β**-**CTX) were detected by the electro**-**chemiluminescence immunoassay method (Roche Cobas, E601 analyzer, Roche Diagnostics, Switzerland). Serum periostin was measured by enzyme**-**linked immunosorbent assay (ELISA) kits (Cat.No. BI**-**20433, BIOMEDICA, Austria. Intra**-**assay CV ≤ 3%, inter**-**assay CV ≤ 6%) as the manufacturer’s protocol guided.

### Muscle function and risk of fall assessment

Muscle function was assessed using handgrip strength, the Short Physical Performance Battery (SPPB) test, and the Timed Up and Go (TUG) test, respectively. Handgrip strength was measured using an electronic handgrip dynamometer (SENSSUM, China). Participants were instructed to stand upright with arms in a neutral position, and handgrip strength was recorded three times for each arm, with the maximum value noted. The SPPB test followed the standard procedure, comprising three tests: the standing balance test, the 2.44**-**meter gait speed test, and the 5**-**time chair stand test (26). Each test was scored from 0 to 4, yielding a total score ranging from 0 to 12. A higher SPPB score indicated better physical performance. The TUG test involved participants being observed and timed as they stood from an armchair, walked 3 meters, turned, walked back, and sat down again (27). Participants completing the TUG test in more than 12 seconds were considered at a high risk of fall; otherwise, they were assessed as having a low risk of fall. The Falls Risk for Older People in the Community (FROP**-**Com) screening tool was also used to evaluate the fall risk of participants (28).

### Dual-energy X-ray absorptiometry assessment of aBMD, lumbar trabecular bone score, and muscle mass

In this study, aBMD, lumbar TBS, and muscle mass of the participants were assessed using a bone density measurement device (Lunar, GE Healthcare, Madison, USA). DXA scans were performed to assess aBMD at three locations in the participants: the total hip, femoral neck, and lumbar spine 1**-**4 (L1**-**4). The absolute bone density values were converted into T**-**scores by comparing the bone mass of participants with peak bone mass of young individuals of the same ethnicity and gender, based on the Chinese reference database provided by GE Lunar. Participants were categorized into normal bone mass (T**-**score ≥ **-**1.0), osteopenia (**-**2.5 < T**-**score < **-**1.0), and osteoporosis based on the lowest T**-**score among the axial bones (total hip, femoral neck, and L1**-**4). Osteoporosis was defined when the T**-**score of axial bones was ≤ **-**2.5 or fragility fractures in the hip or spine or when participants with low bone mass had experienced fragility fractures of the proximal humerus/pelvis/distal forearm.

The data collection process for TBS was similar to that of aBMD but utilized different algorithms and analysis software. TBS values at the lumbar spine were calculated using TBS iNsight v2.1 software (Medimaps).

Body composition analysis was conducted using enCORE 10.50.086 software to obtain appendicular skeletal muscle mass (ASM) and calculate appendicular skeletal muscle mass/height (Ht)^2^. According to the consensus released in 2019 by AWGS, female participants with ASM/Ht^2^ < 5.4 kg/m^2^, handgrip strength < 18kg, along with evidence of decreased physical performance including SPPB score ≤ 9, or 5**-**time chair stand test ≥ 12s, were classified as sarcopenia (29).

### HR-pQCT of the peripheral skeleton

Following the previously outlined protocol, HR**-**pQCT (XtremeCT II scanner, ScancoMedical, Brüttisellen, Switzerland) with a resolution of 61 mm were conducted at the non**-**dominant distal radius and distal tibia of all participants (30). Various HR**-**pQCT parameters were measured, including total area (Tot.Ar), trabecular area (Tb.Ar), cortical area (Ct.Ar), cortical perimeter (Ct.Pm), total volumetric bone mineral density (Tot.vBMD), trabecular volumetric bone mineral density (Tb.vBMD), cortical volumetric bone mineral density (Ct.vBMD), trabecular number (Tb.N), trabecular thickness (Tb.Th), trabecular separation (Tb.Sp), cortical thickness (Ct.Th), cortical porosity (Ct.Po). Estimated bone strength, including stiffness and failure load, was calculated using Scanco Finite Element software (version 1.13; Scanco Medical).

### Statistical analysis

SPSS version 25.0 was used for statistical analysis. The distribution of all continuous variables was determined by the Kolmogorov**-**Smirnov test. Normally distributed continuous variables were depicted as mean ± standard deviation (SD) and non**-**normally distributed continuous variables were shown as median (interquartile range, IQR). Categorical variables were expressed as proportion (counts/sum). Pearson, Spearman and Kendall’s tau**-**b correlation analysis were performed to explore the unadjusted bivariate correlations. Mann**-**Whitney *U* test was used to compare difference between the two groups. The one way analysis of variance (ANOVA) and the Kruskal Wallis H test were used to compare differences of variables in multiple groups according to the distribution of the data. The Chi**-**square test or Fisher’s exact test were used to compare the classified data in multiple groups. Covariance analysis was performed to control possible confounders. Univariate and multivariate linear regression analyses were performed to determine the relationships between serum periostin level and Tot.Ar, Tb.Ar, Ct.Pm, stiffness, failure load, ASM, maximum handgrip strength, the score of the standing balance test, the score of the 2.44**-**meter gait speed test, the score of the 5**-**time chair stand test, the score of the SPPB test, the time of the TUG test and the score of the FROP**-**Com screening tool. *P* value < 0.05 was assigned as a statistically significant difference.

## Results

### Baseline characteristics of the cohort

135 postmenopausal women who had the results of serum periostin were included. The mean age of the participants was 71.8 ± 7.9 years old. The mean of height, weight, BMI were 155.1 ± 6.8 cm, 62.0 ± 11.0 kg, 25.7 ± 4.0 kg/m^2^, respectively. The mean of serum periostin was 1770.3 ± 440.4 pmol/L. The mean of serum Ca, Pi, 25(OH)D were 2.35 ± 0.09 mmol/L, 1.20 ± 0.17 mmol/L, 22.47 ± 9.16 ng/ml, respectively. The medians of serum ALP, iPTH, β**-**CTX, P1NP were 80.0 (71.0, 92.0) U/L, 48.3 (37.6, 63.4) pg/ml, 0.40 (0.30, 0.52) ng/ml, 47.0 (35.0, 60.7) ng/ml, respectively. Detailed characteristics and biochemical parameters are presented in **Table 1**.

**Table 1:** General characteristics among postmenopausal women according to tertiles of serum periostin levels.

	ALL (N = 135)	T1 (n = 45)	T2 (n = 45)	T3 (n = 45)	*P*
General characteristics					
Age (y)	71.8 ± 7.9	71.4 ± 7.1	70.8 ± 7.5	71.0 ± 18.0	0.543
Height (cm)	155.1 ± 6.8	155.8 ± 6.2	156.1 ± 8.3	153.8 ± 6.5	0.082
Weight (kg)	62.0 ± 11.0	64.9 ± 11.4	61.1 ± 10.2	60.0 ± 11.0	0.094
BMI (kg/m2)	25.7 ± 4.0	25.8 ± 6.0	25.1 ± 3.6	25.4 ± 4.0	0.338
Menopausal age (y)	50.0 (47.0, 52.0)	50.0 (48.0, 53.0)	50.0 (47.5, 52.0)	49 (46.0, 51.3)	0.220
Smoking	4.4% (6/135)	2.3% (1/44)	6.7% (3/45)	4.3% (2/46)	0.603
Drinking	3% (4/135)	2.3% (1/44)	2.2% (1/45)	4.3% (2/46)	0.792
Used/using glucocorticoid (≥3 months)	5.2% (7/135)	4.5% (2/44)	8.9% (4/45)	2.2% (1/46)	0.343
VitD and calcium supplementation (≥3 months)	31.1% (42/135)	29.5% (13/44)	33.3% (15/45)	30.4% (14/46)	0.921
HRT (≥3 months)	5.9% (8/135)	9.1% (4/44)	2.2% (1/45)	6.5% (3/46)	0.382
Anti-osteoporotic drugs (≥3 months)	15.6% (21/135)	20.5% (9/44)	13.3% (6/45)	13.0% (6/46)	0.550
Biochemical parameters					
Periostin (pmol/L)	1770.3 ± 440.4	1335.8 ± 323.0	1726.5 ± 96.8	2274.1 ± 214.6	< 0.01
Ca (mmol/L)	2.35 ± 0.09	2.34 ± 0.09	2.34 ± 0.08	2.37 ± 0.10	0.380
Pi (mmol/L)	1.20 ± 0.17	1.18 ± 0.17	1.17 ± 0.21	1.22 ± 0.15	0.417
ALP (U/L)	80.0 (71.0, 92.0)	79.5 (71.0, 91.0)	83.0 (71.0, 96.0)	77.0 (70.0, 92.3)	0.558
iPTH (pg/ml)	48.3 (37.6, 63.4)	49.4 (39.3, 69.0)	49.4 (35.4, 59.2)	47.1 (36.3, 63.6)	0.869
25(OH)D (ng/ml)	22.47 ± 9.16	22.21 ± 6.87	21.40 ± 11.30	19.70 ± 12.40	0.540
β-CTX (ng/ml)	0.40 (0.30, 0.52)	0.37 (0.20, 0.49)	0.44 (0.33, 0.53)	0.41 (0.27, 0.51)	0.134
P1NP (ng/ml)	47.0 (35.0, 60.7)	39.5 (33.6, 56.5)	49.7 (38.9, 60.2)	49.0 (34.8, 62.9).	0.091
ALT (U/L)	18.0 (4.0, 25.0)	18.0 (14.0, 25.8)	16.0 (12.5, 25.5)	18.0 (14.8, 23.5)	0.467
Cr (umol/L)	64.0 (57.0, 76.0)	66.0 (60.0, 78.5)	61.0 (55.0, 67.5)	64.0 (57.8, 78.3)	0.092
aBMD^a^					
L1-4 T-score	-0.57 ± 1.44	-0.45 ± 1.47	-0.44 ± 1.35	-0.80 ± 1.49	0.414
Femoral neck T-score	-1.40 (-2.00, -0.40)	-1.50 (-2.00, -0.73)	-0.90 (-1.70, -0.20)	-1.60 (-2.00, -0.55)	0.164
Total hip T-score	-0.89 ± 1.14	-0.85 ± 1.04	-0.75 ± 1.11	-1.05 ± 1.26	0.373
Percentage of osteoporosis and sarcopenia					
Osteoporosis	23.0% (31/135)	18.2% (8/44)	26.7% (12/45)	8.9% (11/46)	0.148
Sarcopenia	3% (4/135)	0% (0/44)	2.2% (1/45)	6.5% (3/46)	0.178
The risk of fall and the history of fall^a^					
The time of the TUG test (s)	8.2 (7.3, 10.1)	7.6 (7.0, 8.8)	8.1 (7.3, 9.7)	9.5 (7.6, 11.1)	0.017
The score of the FROP-Com screening tool	0.0 (0.0, 1.0)	0.0 (0.0, 1.0)	0.0 (0.0, 1.5)	1.0 (0.0, 2.3)	0.102
The history of fall in recent one year	24.4% (33/135)	22.7% (10/44)	28.9% (13/45)	21.7% (10/46)	0.693
The history of fractures					
The history of fractures after age 50	28.9% (39/135)	27.3% (12/44)	28.9% (13/45)	30.4% (14/46)	0.850
Vertebral fractures	21.5% (29/135)	18.2% (8/44)	24.4% (11/45)	21.7% (10/46)	
The others fractures	16.3% (22/135)	13.6% (6/44)	15.6% (7/45)	19.6% (9/46)	
The history of fractures in recent one year	3.7% (5/135)	4.5% (2/44)	4.4% (2/45)	2.2% (1/46)	0.795

Normally distributed continuous variables were depicted as mean ± standard deviation (SD) and non-normally distributed continuous variables were shown as median (interquartile range, IQR). Categorical variables were expressed as proportion (counts/sum). Normally distributed continuous variables were analyzed by the one way analysis of variance (ANOVA). Non**-**normally distributed continuous variables were analyzed by the Kruskal Wallis H test. The classified data was analyzed by the Chi**-**square test or Fisher’s exact test. ^a^ALL (N = 131), T1 (n = 43), T2 (n = 44), T3 (n = 44). Bold values denoted statistically significant differences (*P* < 0.05).

BMI, body mass index; HRT, hormone replacement therapy; VitD, vitamin D; Ca, serum total calcium; Pi, serum phosphate; ALP, serum alkaline phosphatase; iPTH, serum intact parathyroid hormone; 25(OH)D, 25**-**hydroxy vitamin D; β**-**CTX, C**-**terminal cross**-**linking telopeptide of type I collagen; P1NP, procollagen type 1 N**-**terminal propeptide; ALT, alanine aminotransferase; Cr, serum creatinine; aBMD, areal bone mineral density; L1**-**4, lumbar vertebrae 1**-**4; TUG, Timed Up and Go; FROP**-**Com, the Fall Risk for Older People in the Community.

The mean of T-scores for aBMD of L1-4, total hip were -0.57 ± 1.44 and -0.89 ± 1.14. The median of T**-**score for aBMD of femoral neck was **-**1.40 (**-**2.00, **-**0.40). Among all participants, 31 (23.0%) had osteoporosis. Approximately 3% of participants had sarcopenia.

131 participants from the 135 participants completed the test of TUG. According to the time of TUG, 15 (11.5%) were at high risk of fall, 116 (88.5%) were at low risk of fall. According to the score of the FROP**-**Com screening tool, 11 (8.1%) were at high risk of fall, 124 (91.9%) were at low risk of fall. 33 (24.4%) had history of falls in recent one year. 39 (28.9%) had history of fractures after age 50.

According to the tertiles of serum periostin levels, the participants were divided into three groups. Compared to the T1 group, participants in the T3 had significant longer time of the TUG test. The other characteristics among three groups had no significant differences.

**Figure 1:**
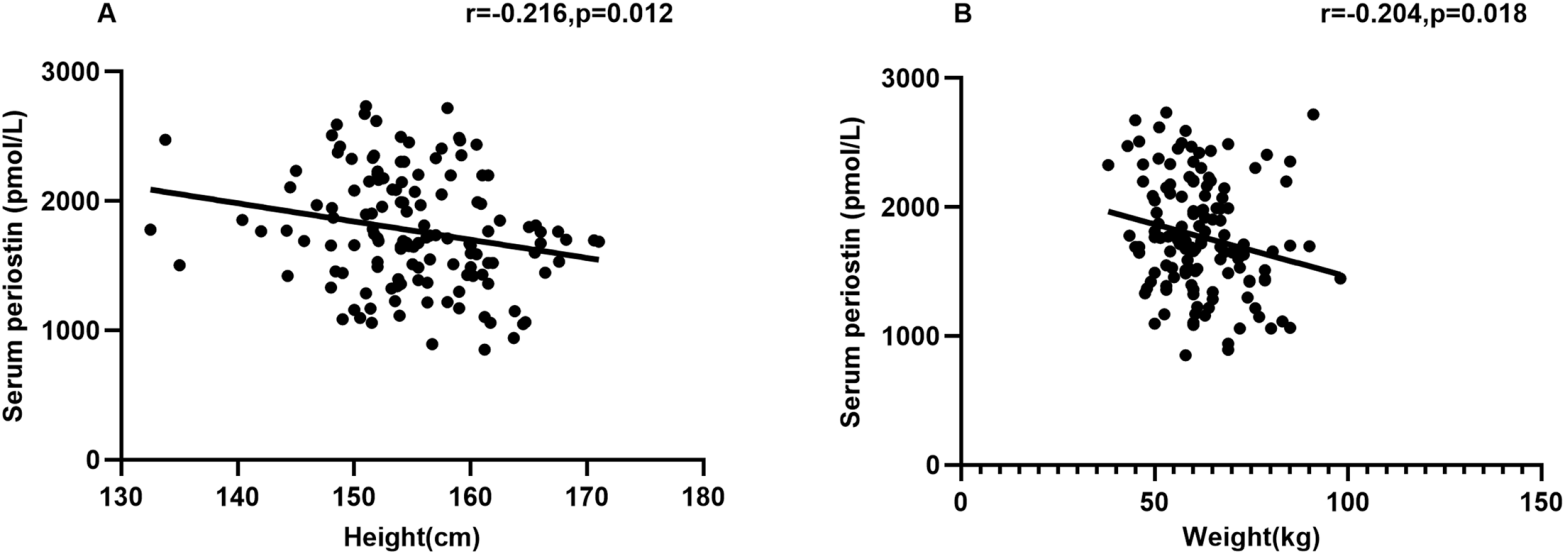
Correlations between serum periostin and height and weight. **(A)** Correlation between serum periostin and height by Pearson analysis. **(B)** Correlation between the serum perisotin and weight by Pearson analysis.

In bivariate analysis, it was observed that serum periostin was negatively correlated with the height (r = **-**0.216, *P* = 0.012) and weight (r = **-**0.204, *P* = 0.018) (**Figure 1**). The associations between serum periostin and biochemical parameters were shown in **Supplementary Table 1**. However, there was no correlation between serum periostin and the biochemical parameters.

### Correlations between serum periostin and aBMD, lumbar TBS and HR-pQCT parameters

**Figure 2:**
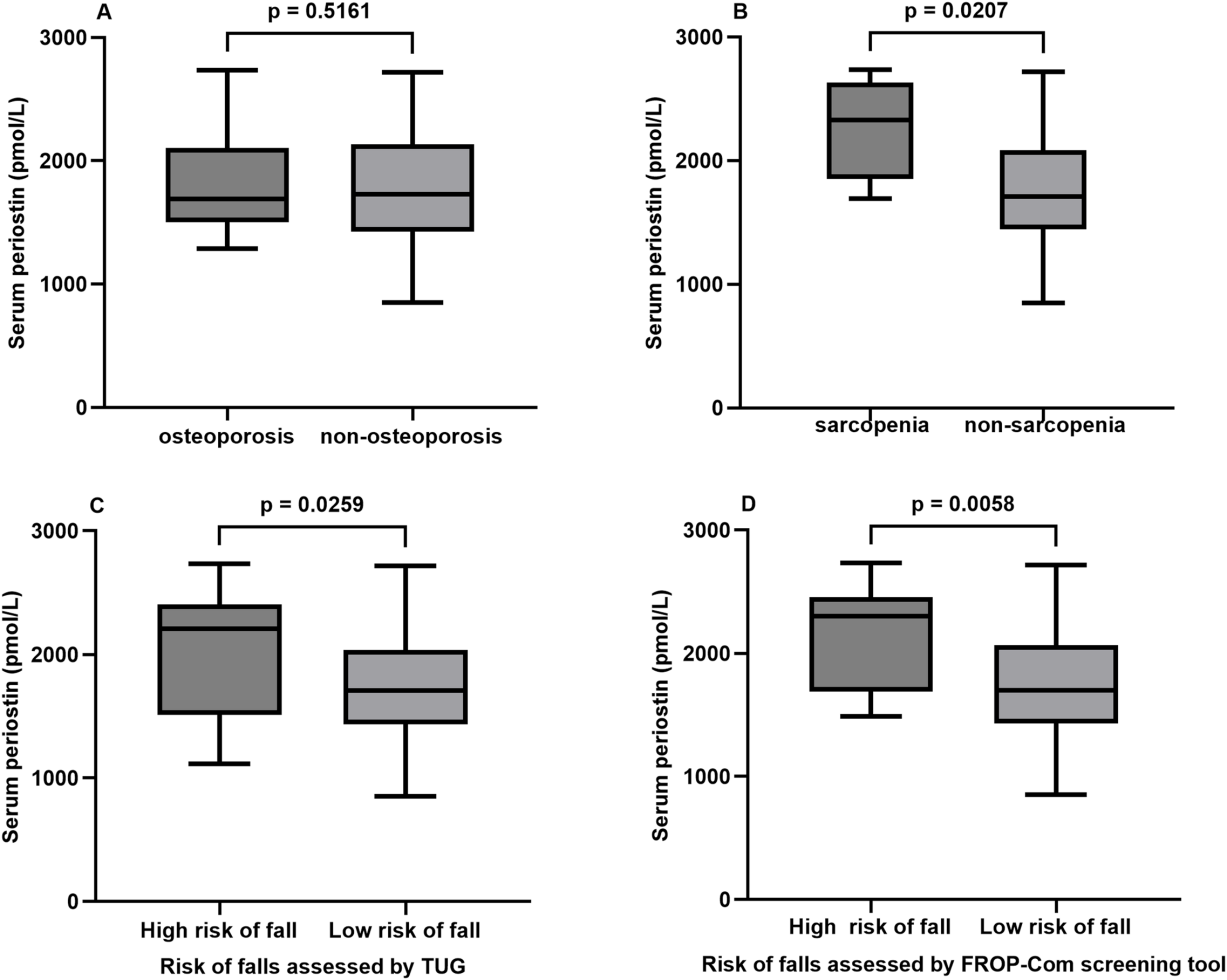
Serum periostin in postmenopausal women with and without **(A)** osteoporosis, **(B)** sarcopenia and risk of fall **(C, D)**. All comparison above was performed by the independent samples t test. TUG, Timed Up and Go; FROP-Com, the Falls Risk for Older People in the Community.

Bivariate correlations between serum periostin and aBMD, lumbar TBS and parameters evaluated by HR**-**pQCT were displayed in **Table 2**. There was no significant correlation between serum periostin with aBMD assessed by DXA at the femoral neck, total hip and L1**-**L4 and lumbar TBS. There was also no significant difference of serum periostin between groups with and without osteoporosis (**Figure 2**).

**Table 2:** Correlation analysis between serum periostin and BMD, lumbar TBS and HR-pQCT parameters.

aBMD						
	BMD			T-score		
	Value	Correlation coefficient	*P*	Value	Correlation coefficient	*P*
L1**-**4	1.055 ± 0.178	**-**0.104	0.237	**-**0.57 ± 1.44	**-**0.107	0.225
Femoral neck	0.750 (0.676, 0.864)	**-**0.033	0.700	**-**1.40 (**-**2.00, **-**0.40)	**-**0.001	0.994
Total hip	0.843 ± 0.143	**-**0.073	0.398	**-**0.89 ± 1.14	**-**0.082	0.343
lumbar TBS						
			correlation coefficient		*P*	
TBS	1.260 ± 0.095		0.118		0.191	
Bone microstructure parameters measured by HR-pQCT						
	Radius			Tibia		
	Value	correlation coefficient	*P*	Value	correlation coefficient	*P*
Bone geometry						
Tot.Ar (mm^2^)	247.7 ± 40.1	-**0.218**	**0.083**	650.3 ± 110.3	-**0.180**	**0.038**
Tb.Ar (mm^2^)	198.8 ± 41.2	-**0.170**	**0.049**	556.7 ± 108.1	**-**0.130	0.135
Ct.Ar (mm^2^)	52.3 ± 10.2	**-**0.146	0.092	101.2 ± 20.1	**-**0.130	0.136
Ct.Pm (mm)	65.8 ± 6.1	-**0.202**	**0.019**	99.4 ± 8.1	-**0.178**	**0.040**
vBMD						
Tot.vBMD (mgHA/cm3)	258.0 (221.9, 304.1)	-0.031	0.724	222.5 (189.0, 270.0)	-0.039	0.654
Tb.vBMD (mgHA/cm3)	92.1 (73.1, 122.0)	-0.083	0.338	118.2 (87.9, 142.2)	-0.082	0.347
Ct.vBMD (mgHA/cm3)	897.3 (85.3, 944.6)	-0.004	0.967	841.9(795.3, 885.7)	0.023	0.788
Bone microarchitecture						
Tb.N (1/mm)	1.128 ± 0.302	-0.077	0.372	1.103 ± 0.241	-0.058	0.507
Tb.Th (mm)	0.218 (0.209, 0.229)	0.017	0.847	0.244 (0.232. 0.260)	-0.077	0.377
Tb.Sp (mm)	0.860 (0.732, 1.095)	0.078	0.369	0.875 (0.782, 1.045)	0.064	0.463
Ct.Th (mm)	0.950 (0.795, 1.061)	-0.037	0.667	1.207 (1.048, 1.422)	-0.044	0.616
Ct.Po	0.007 (0.004, 0.010)	0.012	0.890	0.033 (0.024, 0.045)	-0.018	0.834
Estimated bone strength						
Stiffness (N/mm)	44837.2 ± 11478.4	-0.126	0.145	130770.1 ± 30852.2	-0.213	0.014
Failure load (N)	2381.4 ± 638.5	-0.115	0.184	7159.6 ± 1642.9	-0.199	0.022

Normally distributed continuous variables were depicted as mean ± standard deviation (SD) and non-normally distributed continuous variables were shown as median (interquartile range, IQR). Categorical variables were expressed as proportion (counts/sum). Spearman analysis was used between serum periostin and the other variables. Bold values denoted statistically significant differences (*P* < 0.05).

aBMD, areal bone mineral density; L1**-**4, lumbar vertebrae 1**-**4; TBS, trabecular bone score; HR**-**pQCT, high**-**resolution peripheral quantitative computed tomography; Tot.Ar, total area; Tb.Ar, trabecular area; Ct.Ar, cortical area; Ct.Pm, cortical perimeter; vBMD, volumetric bonemineral density; Tot.vBMD, total vBMD; Tb.vBMD, trabecular vBMD; Ct.vBMD, cortical vBMD; Tb.N, trabecular number; Tb.Th, trabecular thickness; Tb.Sp, trabecular separation; Ct.Th, cortical thickness; Ct.Po, cortical porosity.

Parameters of vBMD at the distal radius and tibia also did not exhibit significant correlations with serum periostin. Serum periostin was negatively correlated with Tot.Ar (radius: r = **-**0.218, *P* = 0.083; tibia: r = **-**0.180, *P* = 0.038) and Ct.Pm (radius: r = **-**0.202, *P* = 0.019; tibia: r = **-**0.178, *P* = 0.040)at the distal radius and tibia, but only negatively correlated with Tb.Ar (r = **-**0.170, *P* = 0.049)at the distal radius. Serum periostin was negatively correlated with estimated bone strength stiffness (r = **-**0.213, *P* = 0.014) and failure load (r = **-**0.199, *P* = 0.022) at the distal tibia.

We performed univariate and multiple linear regression analysis between serum periostin and Tot.Ar and Ct.Pm at the distal radius and tibia further. Univariate and multivariate linear regression analysis were also used to explore the relationship between serum periostin and Tb.Ar at the distal radius and stiffness and failure load at the distal tibia. In univariate linear regression analysis, serum periostin was all negatively correlated with Tot.Ar (radius: standardized β = **-**0.204, adjusted R^2^ = 0.034, *P* = 0.018; tibia: standardized β = **-**0.188, adjusted R^2^ = 0.028, *P* = 0.029) at the distal radius and tibia, Ct.Pm (radius: standardized β = **-**0.174, adjusted R^2^ = 0.023, *P* = 0.043; tibia: standardized β = **-**0.189, adjusted R^2^ = 0.029, *P* = 0.029) at the distal radius and tibia, stiffness (standardized β = **-**0.205, adjusted R^2^ = 0.035, *P* = 0.018) and failure load (standardized β = **-**0.199, adjusted R^2^ = 0.032, *P* = 0.022) at the distal tibia. After adjusting by age, height, weight, there was no correlation between serum periostin with the variable parameters mentioned above (**Table 3**).

**Table 3:** Linear regression analysis between serum periostin and bone, muscle and fall.

Bone									
Radius									
	Unadjusted			Model1			Model2		
	β	R2adj	*P*	β1	R2adj	*P*	β2	R2adj	*P*
Tot.Ar (mm^2^)	-**0.204**	**0.034**	**0.018**	**-**0.233	0.076	0.06	**-**0.133	0.270	0.083
Tb.Ar (mm^2^)	**-**0.160	0.018	0.064	-**0.205**	**0.124**	**0.013**	**-**0.124	0.249	0.111
Ct.Pm (mm)	-**0.174**	**0.023**	**0.043**	-**0.205**	**0.069**	**0.016**	**-**0.111	0.240	0.156
Tibia									
	Unadjusted			Model1			Model2		
	β	R^2^adj	*P*	β1	R^2^adj	*P*	β2	R^2^adj	*P*
Tot.Ar (mm^2^)	-**0.188**	**0.028**	**0.029**	-**0.203**	**0.031**	**0.020**	**-**0.073	0.404	0.291
Ct.Pm (mm)	-**0.189**	**0.029**	**0.029**	-**0.202**	**0.029**	**0.021**	**-**0.063	0.457	0.339
Stiffness (N/mm)	-**0.205**	**0.035**	**0.018**	**-**0.134	0.249	0.081	**-**0.059	0.363	0.417
Failure load (N)	**-0.199**	**0.032**	**0.022**	**-**0.165	0.028	0.098	**-**0.077	0.044	0.483
Muscle									
	Unadjusted			Model1			Model2		
	β	R^2^adj	*P*	β_1_	R^2^adj	*P*	β_2_	R^2^adj	*P*
ASM (kg)	-**0.211**	**0.037**	**0.014**	-**0.177**	**0.095**	**0.035**	**-**0.008	0.791	0.851
Maximum handgrip strength (kg)	**-**0.161	0.018	0.063	-**0.379**	**0.154**	**< 0.001**	**-**0.650	0.215	0.517
The score of the standing balance test	**-**0.162	0.019	0.061	**-**0.110	0.164	0.170	**-**0.110	0.153	0.183
The score of the 2.44**-**meter gait speed test	-**0.271**	**0.066**	**0.001**	-**0.230**	**0.154**	**0.005**	-**0.242**	**0.146**	**0.004**
The score of the 5**-**time chair stand test	0.053	**-**0.005	0.546	**-**0.013	0.207	0.868	0.033	0.244	0.675
The score of the SPPB test	-**0.220**	**0.041**	**0.010**	-**0.165**	**0.202**	**0.035**	-**0.190**	**0.204**	**0.018**
The time of the TUG test (s)	**0.274**	**0.068**	**0.002**	**0.215**	**0.228**	**0.007**	**0.237**	**0.225**	**0.004**
The score of the FROP**-**Com screening tool	**0.250**	**0.055**	**0.003**	**0.239**	**0.049**	**0.012**	**0.232**	**0.078**	**0.014**

Model 1, adjusted by age; Model 2, adjusted by age, height, weight. “β, β_1_, and β_2_” were standardized b value. R^2^adj, adjusted r**-**square. Bold values denoted statistically significant differences (*P* < 0.05).

Tot.Ar, total area; Tb.Ar, trabecular area; Ct.Pm, cortical perimeter; ASM, appendicular skeletal muscle mass; SPPB, Short Physical Performance Battery; TUG, Timed Up and Go; FROP-Com, the Falls Risk for Older People in the Community.

### Correlations between serum periostin and muscle mass, muscle function and risk of fall

The associations between serum periostin and muscle mass, muscle function and risk of fall were shown in **Table 4**. Serum periostin was negatively correlated with ASM (r = **-**0.229, *P* = 0.008). Serum periostin was negatively correlated with the standing balance test score (r = -0.178, P = 0.038) and the 2.44-meter gait speed test score (r = -0.238, P = 0.005). Serum periostin was positively correlated with the time of TUG test (r = 0.281, *P* = 0.001) and the score of the FROP**-**Com screening tool (r = 0.181, *P* = 0.036).

**Table 4:** Correlation analysis between serum periostin and muscle, fall and fractures.

	Value	correlation coefficient	*P*
Muscle mass			
ASM (kg)	14.9 (13.6, 16.4)	-**0.229**	**0.008**
ASM/Ht^2^ (kg/m^2^)	6.19 (5.70, 6.72)	**-**0.104	0.228
Muscle function			
Maximum handgrip strength (kg)	21.4 ± 4.9	**-**0.155	0.072
The score of the standing balance test	4.0 (4.0, 4.0)	-**0.178**	**0.038**
The score of the 2.44**-**meter gait speed test	4.0 (4.0, 4.0)	-**0.238**	**0.005**
The score of the 5**-**time chair stand test	4.0 (3.0, 4.0)	**-**0.073	0.400
The score of the SPPB test	12.0 (11.0, 12.0)	**-**0.152	0.079
Risk of fall			
The time of the TUG test (s)	8.2 (7.3, 10.1)	**0.281**	**0.001**
The score of the FROP**-**Com screening tool	0.0 (0.0, 1.0)	**0.181**	**0.036**
**The history of fall in recent one year**	24.4% (33/135)	**-**0.013	0.858
**The history of fractures after age 50**	28.9% (39/135)	0.022	0.796
**The history of fractures in recent one year**	3.7% (5/135)	**-**0.075	0.289

Normally distributed continuous variables were depicted as mean ± standard deviation (SD) and non-normally distributed continuous variables were shown as median (interquartile range, IQR). Categorical variables were expressed as proportion (counts/sum). Spearman analysis and Kendall’s tau**-**b analysis were used between serum periostin and the other variables. Bold values denoted statistically significant differences (*P* < 0.05).

ASM, appendicular skeletal muscle mass; Ht: height; SPPB, Short Physical Performance Battery; TUG, Timed Up and Go; FROP**-**Com, the Falls Risk for Older People in the Community.

In univariate linear regression analysis, serum periostin had negative correlations with ASM (standardized β = **-**0.211, adjusted R^2^ = 0.037, *P* = 0.014), the score of the 2.44**-**meter gait speed test (standardized β = **-**0.271, adjusted R^2^ = 0.066, *P* = 0.001) and the score of the SPPB test (standardized β = **-**0.220, adjusted R^2^ = 0.041, *P* = 0.010) and had positive correlations with the time of the TUG test (standardized β = 0.274, adjusted R^2^ = 0.068, *P* = 0.002) and the score of the FROP**-**Com screening tool (standardized β = 0.250, adjusted R^2^ = 0.055, *P* = 0.003).

After adjusting by age, height, weight, serum periostin was still negatively correlated with the 2.44**-**meter gait speed test (standardized β = **-**0.242, adjusted R^2^ = 0.146, *P* = 0.004) and the score of the SPPB test (standardized β = **-**0.190, adjusted R^2^ = 0.204, *P* = 0.018) and positively correlated with the time of the TUG test (standardized β = 0.237, adjusted R^2^ = 0.225, *P* = 0.004) and the score of the FROP**-**Com screening tool (standardized β = 0.232, adjusted R^2^ = 0.078, *P* = 0.014) (**Table 3**).

Participants with sarcopenia had significantly higher serum periostin level than those without sarcopenia (*P* = 0.0207). However, after adjusting age, height, weight, there was no significant difference between the two groups (*P* = 0.071). Participants with high risk of fall had significantly higher serum periostin level than those with low risk of fall assessed by the time of TUG test and the score of the FROP**-**Com screening tool (TUG: *P* = 0.0259; FROP**-**Com screening tool: *P* = 0.006). After adjusting age, height, weight, difference between the two groups assessed by the time of TUG test was no longer significant (*P* = 0.069), while difference between the two groups assessed by the score of the FROP**-**Com screening tool was still significant (*P* = 0.013) (**Figure 2**).

### Correlations between serum periostin and the fractures

Bivariate correlations between serum periostin and the history of the fractures were shown in **Table 4**. However, there was no correlation between serum periostin with the history of the fractures.

## Discussion

This study was the first attempt to simultaneously explore the relationship between serum periostin and bone microarchitecture, muscle mass and function in postmenopausal women. In this study, we found that serum periostin may be the independent predictor of the muscle function in lower limbs and risk of fall.

This study did not find the correlations between serum periostin level and central aBMD and lumbar TBS measured by DXA. In populations with characteristics similar to those included in our study—namely, community-based or naturally recruited cohorts of Chinese postmenopausal women, no significant association between serum periostin and aBMD was observed (14, 16, 17). In contrast, in patients with primary hyperparathyroidism, osteopenia, or osteoporosis, periostin was reported to be negatively correlated with aBMD at specific skeletal sites (15, 18). These findings suggested that the discrepancy across studies largely reflected differences in study populations. Future studies with more rigorous designs, well-defined populations, and stratified analyses were warranted to clarify the true nature and clinical significance of the relationship between periostin and aBMD. Further, in HR-pQCT analysis, we didn’t find correlation between serum periostin and bone vBMD but identified negative correlations between serum periostin and Tot.Ar and Ct.Pm at the distal radius and tibia, and a negative correlation with Tb.Ar at the distal radius. We also found negative correlations between serum periostin and estimated bone strength stiffness and failure load at the tibia. A previous study involving healthy individuals did not found correlation between serum periostin and Ct.Pm at the distal radius and tibia (31). The findings of this study differed from the previous report, which may have been due to differences in the study populations. The specific relationship between periostin and bone required further investigation in prospective studies.

In addition, we found that serum periostin was negatively correlated with ASM. Higher periostin levels were also associated with poorer performance on functional tests, including lower scores on the standing balance test and slower 2.44-meter gait speed. Moreover, serum periostin was positively correlated with longer time of the TUG test and higher scores on the FROP-Com screening tool, indicating a potential link between elevated periostin levels and increased fall risk.

Consistent with our findings, a study of 1,096 older adults reported that individuals with higher serum periostin levels experienced a greater annual decline in SPPB scores over a 4-year follow-up compared with those with lower levels (32). These clinical observations were also in line with experimental evidence. Two studies in Postn^-^/^-^ mice showed reduced skeletal muscle fibrosis, along with significant improvements in muscle structure and function (23, 24). Taken together, these findings suggest that elevated periostin levels may promote muscle fibrosis, thereby contributing to impaired skeletal muscle health and functional decline (23, 24).

Furthermore, several previous studies have reported a relationship between serum periostin and osteoporotic fracture risk. Kim et al. found that higher serum periostin levels might indicate a higher risk of osteoporotic fractures, particularly at non**-**vertebral sites (20). Rousseau and colleagues conducted a prospective cohort study revealing that higher baseline serum periostin levels were correlated with an increased risk of future osteoporotic fractures in postmenopausal women (19). However, a study in China did not find a correlation between serum periostin and vertebral fracture risk, consistent with our findings (15). The discrepancies between our findings and those of previous studies are likely attributable to several factors: first, differences in the study populations; second, fracture history in our study was obtained via questionnaires and may be subject to recall bias; and third, the overall prevalence of fractures in this cohort was relatively low. Further research is needed to reconcile these findings and better understand the role of periostin in fracture risk.

This study still had some limitations. Firstly, the number of participants in this study was limited, which restricted further exploration. Secondly, this study was a cross-sectional study that did not include follow-up, so causal conclusions could not be drawn. Thirdly, the history of falls was obtained through questionnaires in this study, which might have recall bias, affecting the further exploration of the relationship between serum periostin and fall. Fourthly, although severe vitamin D deficiency was rare in this cohort, it was not completely excluded, which could potentially influence bone metabolism and periostin levels. To further clarify whether serum periostin level can serve as a marker for assessing the function of the musculoskeletal system in postmenopausal women, we should increase the number of participants to conduct a prospective study in the future.

In conclusion, elevated serum periostin levels in postmenopausal women were predominantly associated with impaired muscle function and increased fall risk. After multivariable adjustment, the associations with bone geometry, bone strength, and muscle mass were no longer significant. These findings indicate that periostin may represent a biomarker of musculoskeletal functional status, particularly muscle performance, in postmenopausal women. Further prospective studies are required to validate these observations.

## Data Availability

The raw data supporting the conclusions of this article will be made available by the authors, without undue reservation.
