# Supplementary material for: Association of serum periostin with bone microarchitecture, muscle mass and function in Chinese postmenopausal women
Source: Front Endocrinol (Lausanne). 2026 Apr 7;17:1808866. doi: 10.3389/fendo.2026.1808866 (PMC13095588; doi:10.3389/fendo.2026.1808866)
Supplement: Supplementary file 1 [file Table1.docx]

**Supplement Table 1. Correlation analysis between serum periostin and biochemical parameters**

|  | r | *P* |
| --- | --- | --- |
| Ca (mmol/L) | 0.091 | 0.296 |
| Pi (mmol/L) | 0.061 | 0.479 |
| ALP (U/L) | **-**0.003 | 0.973 |
| iPTH (pg/ml) | **-**0.066 | 0.444 |
| 25(OH)D (ng/ml) | **-**0.001 | 0.991 |
| β**-**CTX (ng/ml) | 0.045 | 0.604 |
| P1NP (ng/ml) | 0.121 | 0.162 |
| Cr (umol/L) | **-**0.037 | 0.671 |

Normally distributed continuous variables were analyzed by Pearson analysis. Non**-**normally distributed continuous variables were analyzed by Spearman analysis. Bold values denoted statistically significant differences (*P* < 0.05).

Abbreviations: Ca, serum total calcium; Pi, serum phosphate; ALP, serum alkaline phosphatase; iPTH, serum intact parathyroid hormone; 25(OH)D, 25**-**hydroxy vitamin D; β**-**CTX, C**-**terminal cross**-**linking telopeptide of type I collagen; P1NP, procollagen type 1 N**-**terminal propeptide; Cr, serum creatinine.
